# Paraneoplastic Syndromes in Gallbladder Cancer: A Systematic Review

**DOI:** 10.3390/medicina61030417

**Published:** 2025-02-27

**Authors:** Beth Shin Rei Lau, Nevin Yi Meng Chua, Wee Teck Ong, Harjeet Singh, Vor Luvira, Kyoichi Takaori, Vishal G. Shelat

**Affiliations:** 1 Lee Kong Chian School of Medicine, Nanyang Technological University, Singapore 308232, Singapore; nchua013@e.ntu.edu.sg (N.Y.M.C.); ongw0109@e.ntu.edu.sg (W.T.O.); vishal_g_shelat@ttsh.com.sg (V.G.S.); 2 Department of GI Surgery, HPB and Liver Transplant, PGIMER Chandigarh, Chandigarh 160012, India; harjeetsingh1982@gmail.com; 3 Department of Surgery, Faculty of Medicine, Khon Kaen University, Khon Kaen 40002, Thailand; vor@kku.ac.th; 4 Pancreatic Cancer Unit, Kyoto University Hospital Cancer Center, Kyoto 606-8507, Japan; takaori@iasgo.org; 5 Department of General Surgery, Tan Tock Seng Hospital, Singapore 308433, Singapore

**Keywords:** gallbladder cancer, paraneoplastic syndrome, leukocytosis, dermatomyositis, polymyositis, acanthosis nigricans, Sweet’s syndrome, exfoliative dermatitis, hypercalcaemia, hyponatremia

## Abstract

*Background and Objectives*: Gallbladder cancer (GBC) is a biologically aggressive malignancy characterised by poor survival outcomes often attributed to delayed diagnosis due to nonspecific clinical presentations. Paraneoplastic syndromes (PNSs), atypical symptoms caused by cancer itself, may serve as valuable indicators for timely diagnosis, particularly in malignancies with nonspecific features. Understanding the manifestations of PNSs in GBC is, therefore, critical. This systematic review collates case studies documenting the association of PNS with GBC, including subsequent management and clinical outcomes. *Materials and Methods*: A comprehensive search of PubMed, Embase, CINAHL, Web of Science, and Cochrane Library databases yielded 49 relevant articles. Upon searching other information sources, two more relevant articles were identified via citation sources. *Results:* The paraneoplastic syndromes were classified according to haematological (leukocytosis), dermatological (inflammatory myositis like dermatomyositis and polymyositis, acanthosis nigricans, Sweet’s syndrome, exfoliative dermatitis), neurological, metabolic (hypercalcemia, hyponatremia), and others (chorea). The analysis included the age, sex, and country of origin of the patient, as well as the time of PNS diagnosis relative to GBC diagnosis. Furthermore, common presenting complaints, investigations, and effectiveness of treatment modalities using survival time were assessed. *Conclusions*: While PNS management can offer some benefits, oncologic outcomes of GBC are largely poor. The majority of PNS in GBC are reported in advanced stages, and, hence, PNS has a minimal role in early diagnosis. PNS management can improve a patient’s quality of life, and thus recognition and treatment are important considerations in the holistic management of GBC patients.

## 1. Introduction

Primary gallbladder cancer (GBC) is a rare but lethal malignancy, constituting 1.2% of all cancers worldwide and 1.7% of cancer-related deaths [1,2]. Most GBCs progress silently and often present at a late stage, with a mean survival rate of 6 months and a 5-year survival rate of 5% [3,4]. As such, early diagnosis is crucial to improve mortality and morbidity [5]. GBC has a rather non-specific clinical presentation, showing vague symptoms such as anorexia and weight loss, as a precursor to jaundice, which would happen at a late stage with hepatic invasion or biliary tree compression from nodal metastasis.

GBC has significant geographic and demographic variation in its epidemiology. The highest incidence rates are reported in parts of Asia and South America, particularly in Chile, India, and China, whereas Western countries, such as the United States and most of Europe, exhibit a lower prevalence [6]. The disease is more common in women, with a female-to-male ratio of approximately 3:1, and is typically diagnosed in individuals over the age of 60 [3]. Several risk factors are strongly associated with GBC, including chronic gallstone disease, which is present in up to 80% of cases, and porcelain gallbladder, a condition characterized by calcification of the gallbladder wall. Other conditions linked to GBC include chronic inflammation due to infections such as Salmonella typhi, obesity, and primary sclerosing cholangitis. Additionally, genetic predispositions and environmental exposures, such as the high consumption of processed foods and industrial carcinogens, have been implicated in disease development [7].

On rare occasions, GBC can be associated with paraneoplastic syndromes (PNSs)—most commonly dermatomyositis, leukocytosis, neurological syndromes and metabolic disturbances such as hypercalcaemia and hyponatremia [8]. While these syndromes are linked to cancer, the exact reason why they occur is not fully understood. Often, these symptoms are mistaken for other illnesses until the cancer is discovered, sometimes at a late or advanced stage. Physicians must be vigilant regarding the telltale signs of PNS and consider GBC in their diagnostic differentials. This systematic review offers a detailed exploration of the PNS linked to primary GBC and their impact on the diagnosis, management, and clinical outcomes.

A previous narrative review has already been conducted by Ali et al. [9] on the spectrum of paraneoplastic syndromes associated with gallbladder carcinoma. However, in the study, Ali only focused on PNS linked with gallbladder carcinoma and not gallbladder cancer in general. The pathophysiology of the PNS was not addressed, and there was also no mention of the relevant investigations linked to the respective PNS or management strategies adopted to address the symptoms. Two previous literature reviews were conducted by Tohyama et al. [10] and Wataru Izumo et al. [11], which explored the extent of leukocytosis as a paraneoplastic syndrome in gallbladder cancers. However, both of these studies only focused on leukocytosis as the sole paraneoplastic syndrome. The link between leukocytosis and granulocyte-colony stimulating factor is also not further explored or searched for. This study bridges that problem by accounting for and explaining the relationship between the two. This study also adopts a systematic procedure by employing a more robust search strategy as well as assessing the risk of bias in the studies used.

This systematic review is the first to comprehensively evaluate the spectrum of PNSs in GBC, bridging key knowledge gaps in their pathophysiology, diagnosis, and clinical impact. Unlike prior reviews that focused on isolated manifestations, this study systematically categorises PNSs into haematological, dermatological, neurological, metabolic, and other subtypes, providing a broader and more detailed perspective. By employing a rigorous search strategy and assessing the risk of bias in included studies, this article strengthens the understanding of PNSs as potential diagnostic clues and prognostic indicators in GBC. Given the aggressive nature and late-stage detection of GBC, recognizing PNS could facilitate earlier diagnosis and optimize patient management. This study underscores the importance of heightened clinical vigilance for PNSs in GBC and lays the groundwork for future research on their pathophysiological mechanisms and therapeutic implications.

## 2. Materials and Methods

### 2.1. PICO Question

This study focused on adult patients (≥18 years old) with a confirmed diagnosis of GBC using imaging. The primary interest is in patients presenting with PNS, meaning those exhibiting symptoms not typically observed in the majority of GBC patients. To explore this unique patient population, a variety of study types, including case reports, case studies, clinical trials, and randomised controlled trials were analysed. PNS was defined as a systemic clinical manifestation of a malignancy causing an altered immune system [12].

### 2.2. Prisma Statement

This review adhered to the reporting guidelines of Preferred Reporting Items for Systematic Reviews and Meta-analyses (PRISMA). 

### 2.3. Exclusion Criteria

The criteria for exclusion included studies reporting on patients below 18 years of age, letters (of any form, including letters to editors), non-English studies, editorials, animal studies, and cancers that were deemed to be non-primary GBC.

### 2.4. Information Sources

A comprehensive literature search was conducted in five medical databases (PubMed, Embase, CINAHL, Web of Science, and Cochrane Library) up to April 2024, utilising MeSH terms and keywords. The search aimed to identify common patterns of PNS associated with GBC. Commonly reported symptoms included leukocytosis (often associated with elevated G-CSF (granulocyte colony-stimulating factor) levels), dermatomyositis, hyponatremia, and hypercalcaemia. These symptoms were incorporated into the search strategy following an initial broad search using “paraneoplastic” as a key phrase. Due to the limited research on this specific subject, the majority of studies identified were case studies. No case series or systematic reviews were found. Consequently, a forest plot and meta-analysis were not feasible.

### 2.5. Database Refinement

The search results were exported and collated using the EndNote library. Duplicate records were removed using the software’s duplicate removal function. The remaining 1022 articles were then imported into Covidence, a systematic review management software, for manual screening. Two review authors independently screened the records using a dual-author, double-blinded method, reviewing titles and abstracts based on pre-defined inclusion and exclusion criteria. Covidence was employed as the screening platform. The University of Oxford Centre for Evidence-Based Medicine’s criteria were used to critically appraise the shortlisted references and determine their reliability and applicability. The full text of potentially eligible references was then retrieved and independently assessed by two review authors, with the assistance of a third review author to resolve any disagreements or uncertainties. The reasons for excluding ineligible studies were meticulously documented.

Following the initial duplicate removal process, a total of 1022 papers remained. These were sourced from various databases: 397 from PubMed, 29 from Cochrane, 377 from Embase, 10 from CINAHL, and 209 from Web of Science.

### 2.6. Data Selection

Data from each included article were systematically extracted and compiled using a standardised template and then organised into a table format within Microsoft Excel. The data table included the article information (first author, year published, study design, article title, country), patient characteristics (overall sample size, study population, male:female ratio, mean/median age, GBC confirmatory diagnosis method, GBC type, presenting symptom, other highlighted comorbidities), PNS features (timing of PNS diagnosis, characteristics of gallbladder tumour, main PNS, associated symptoms), risk of study bias (this field documented the assessed risk of bias using a standardised tool), and study outcomes including metabolic (serum sodium, serum osmolality, urine osmolality, urine sodium, corrected calcium), dermatologic (erythrocyte sedimentation rate, leukocyte count), and hematologic (highest reported white blood cell count, laboratory G-CSF count). Any additional tests, management strategies, and survival time were reported.

### 2.7. Bias Assessment

The risk of bias in the included articles was assessed using the criteria specified in the Joanna Briggs Critical Appraisal Tool for Case Reports [13]. This included an assessment of the following:

1. A clear description of the patient’s demographic and clinical characteristics (e.g., age, gender, medical history, etc.);
2. Detailed description of the patient’s condition or diagnosis;
3. Intervention or treatment provided and any changes made during the course of treatment;
4. Clinical outcomes (improvement, complications, or other changes following the intervention);
5. Follow-up and the duration of observation post-intervention;
6. Whether there were any adverse events anticipated or identified;
7. Discussion of why the case was unique or significant;
8. Clear conclusions drawn from the case, including implications for future clinical practice or research.

Each study was assessed with a score out of 8. Studies with lower scores implied the presence of bias in them. Two review authors independently used the criteria above to assess bias and resolved any differences by discussion with a third reviewer. Biasedness was summarised along with the characteristics of the included studies, which can be seen from the tables below.

### 2.8. Main Results

A total of 49 studies were included in the manuscript. The majority of the studies included were case reports. The outcomes extracted were as follows.

Primary outcomes

- Development of any PNS post diagnosis of GBC;
- Type of treatments for GBC and PNS and its response.

Secondary outcomes

- Investigation findings used to determine the presence of PNS;
- Signs or symptoms associated with the PNS.

**Figure 1:**
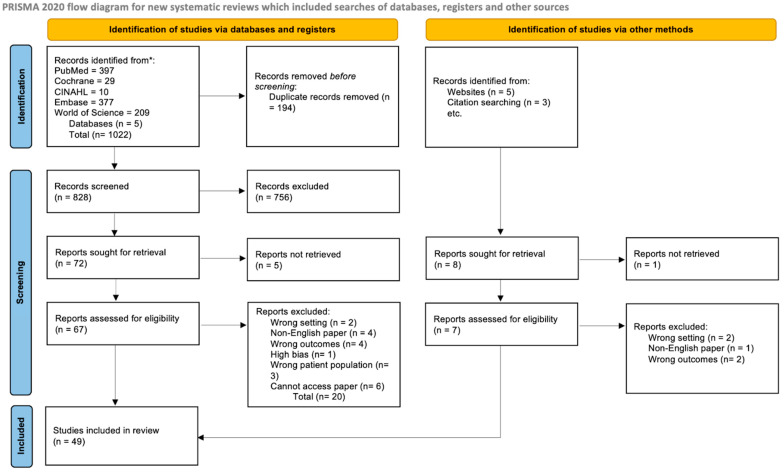
PRISMA chart showing extraction of studies. * Records were screened and identified by authors B.S.R.L. and W.T.O. Any conflicts were resolved by author N.Y.M.C.

The study extraction process is described in our PRISMA-P chart, as shown in Figure 1.

## 3. Results

### 3.1. Haematological Paraneoplastic Syndromes

#### 3.1.1. Leukocytosis

##### Pathophysiology

Leukocytosis, defined as a white blood cell count above 11,000/µL [14], is a common PNS. In a review of GBC cases, leukocytosis was present in 34% of the cases, commonly attributed to tumour necrosis and inflammatory cytokine production [15,16]. It affects about 20% of solid tumour patients [17], with G-CSF found to be the most implicated cytokine [18]. G-CSF promotes myeloid progenitor proliferation, leading to neutrophilia and a pro-inflammatory state via IL-1, IL-6, and TNF-α [19]. Tumour-derived G-CSF was further found to be linked to angiogenesis, metastasis, and systemic inflammatory signs such as low-grade fever.

Leukocytosis in gallbladder cancer (GBC) can arise from three main sources: (1) paraneoplastic leukocytosis (PL), (2) metastatic leukocytosis, and (3) infection-related leukocytosis. Paraneoplastic leukocytosis is characterised by extreme leukocytosis without bone marrow infiltration. It is primarily cytokine-driven, with G-CSF and IL-6 being the main contributors. Other causes of leukocytosis, such as metastatic leukocytosis, typically present with a moderate WBC elevation (12,000–20,000/μL) and bone marrow abnormalities [20], while infectious leukocytosis, such as in cholangitis, is accompanied by fever, positive blood cultures, and typically resolves with antibiotic treatment, unlike PL. Persistent elevation of WBC count without evidence of infection suggests PL, while bone marrow examination can be considered to help differentiate PL from metastatic involvement. Among the reviewed studies, PL was the most likely form of leukocytosis observed in GBC.

##### Results

Fifteen studies met the inclusion criteria, comprising 15 patients (8 females, 7 males) with a mean age of 60.1 years (range: 40–79). The results of the studies is summarised in Table 1. The mean leukocyte count was 42,000/µL (range: 12,600–132,000/µL). Five patients had extreme leukocytosis (>50,000/µL), meeting the criteria for Paraneoplastic Leukemoid Reaction (PLR), a severe form of leukocytosis linked to aggressive tumour biology. A total of 11 out of 15 patients also presented with a concurrent increase in serum G-CSF levels. The average G-CSF value was 286.9 pg/mL (range: 54–800), above the average G-CSF levels (<30 pg/mL) found in healthy individuals [21]. The first case of G-CSF linked to malignancy was reported by Asano [22]. To objectively classify G-CSF-producing cancer, Asano proposed four diagnostic criteria: (a) leukocytosis linked to malignancy that cannot be better explained by other causes, (b) elevated serum G-CSF levels, (c) a decrease in leukocyte and serum G-CSF count post tumour resection, and finally, and (d) immunohistological confirmation of G-CSF production within the tumour. Fulfilment of any single criterion strongly suggests that the cancer is G-CSF-producing. It should be noted that the sensitivity of criterion (d) is not high, with studies indicating it was only approximately 70% sensitive [23,24]. Using criteria (a)–(c), all 11 patients with elevated G-CSF levels can be assumed to be very likely to have a G-CSF-producing GBC.

**Figure 2:**
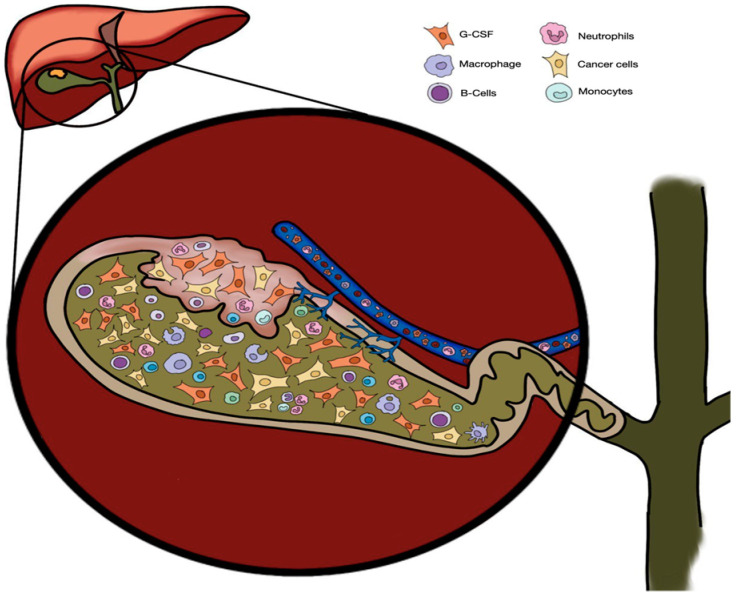
Paraneoplastic leukocytosis of gallbladder carcinomas entering the bloodstream through venous outflow.

The aetiology of the nonspecific abdominal pain may be attributed to chronic cholecystitis or gallbladder wall thickening [25]. Interestingly, some patients with confirmed GBC also displayed concomitant cholecystitis on CT scans. While the precise correlation between cholecystitis and GBC remains unclear, a proposed mechanism involves recurrent acute cholecystitis, leading to the release of pro-inflammatory cytokines such as IL-1, IL-6, and tumour necrosis factor-alpha (TNF-α). These cytokines may create a conducive environment for cancer development [26], as seen in Figure 2. Additionally, chronic mucosal inflammation associated with longstanding gallstones is considered a risk factor for carcinogenesis. Due to nonspecific symptoms, the majority of GBCs were discovered at a locally advanced stage [27]. This, coupled with severe leukocytosis and PLR, often leads to diagnostic confusion between infection and underlying cancer.

**Table 1:** Studies reporting paraneoplastic leukocytosis associated with gallbladder cancer (GBC).

S/N	First Author, Year	Age/Sex	Country	Timing of PNS Diagnosis	Type of Cancer	Tumour Characteristic	Presentation	Investigations	Management	Survival Time After PNS Diagnosis	Study Bias
1	Taiji Tohyama, 2023 [10]	68/F	Japan	ATD	Squamous cell carcinoma of the Gallbladder	M ^d^	-	Neutrophilia Transaminitis Enlarged lymph nodes	Hepatic resection Regional lymph node dissection with extrahepatic bile duct resection Hepatocholangiojejunostomy	Alive	8
2	Y Watanabe, 1989 [11]	54/F	Japan	ATD	Adenocarcinoma	M	Remittent Fever, Left hypochondriac pain	CT abdo: gallstones, round mass in gb, multiple low-density areas in liver lobes Repeat FBC Neutrophilia High segmented WBC	Systemic chemotherapy with doxorubicin and tegafur	47 days	8
3	Muhammad Usman A Khan, 2023 [16]	40/M	Pakistan	ATD	Adenosquamous carcinoma of the gallbladder	LA	RHC Pain, positive Murphy’s	Neutrophilia Lymphadenopathy Multiple GB stones in the lumen	Open cholecystectomy through Kocher’s incision	25 days	8
4	Nobuhiko Kanaya, 2016 [19]	47/F	Japan	ATD	Undifferentiated carcinoma with spindle and giant cells and papillary adenocarcinoma	LA	Fever, general fatigue, 3 kg weight loss	Thrombocytopenia, neutrophilia, C-reactive: 17.27, ALT ^f^:29, ALP ^g^:1017, IL2: 2290	Resection of hepatic S4a + S5 and regional lymph node dissection, along with partial resection of the duodenum	Alive	7
5	Tatsuya Kato, 2002 [28]	70/M	Japan	ATD ^a^	malignant fibrous histiocytoma	LA ^c^	Right-sided upper abdominal mass	Neutrophilia Transaminitis Elevated CRP ^e^	Laparatomy with tumor and gallbladder en bloc excision as well as part of the liver bed	Alive	7
6	Masashi Tsunematsu, 2019 [29]	48/M	Japan	ATD	Not stated	LA	Motor speech disorders, difficulty in movement of both hands, abdominal distension due to hepatomegaly, right upper quadrant pain, fever, systemic edema, loss of appetite and general malaise	Elevated CA19-9	Anticoagulation therapy and systemic chemotherapy [gemcitabine (1000 mg/m^2^) and cisplatin (25 mg/m^2^) given on days 1 and 8	15 days	8
7	Kazunori Shimada, 2009 [30]	69/M	Japan	ATD	Carcinosarcoma	LA	Right upper quadrant abdominal pain, fever	High AFP ^h^ Transaminitis Immunohistochemical cytokeratin positive	Cholecystectomy, resection of the liver bed, and lymph node dissection as a radical operation	Alive	7
8	Mutsuo Furihata, 2002 [31]	48/F	Japan	AD ^b^	Adenocarcinoma	M	Fever and RUQ pain	Granulocytosis, High LDH ^i^, Elevated tumour markers	Cholecystectomy with partial right lobectomy of liver	Not stated	8
9	Wataru Izumo, 2016 [32]	67/M	Japan	ATD	Adenocarcinoma	M	Fever	Elevated ALP and y-GTP ^j^ Elevated CRP	Cholecystectomy, partial resection of segments 4 and 5 of the liver, and partial resection of the transverse colon) and gastrostomy	Alive	8
10	M Takahashi, 1985 [33]	72/F	Japan	AD	Adenocarcinoma	LA	Right upper abdominal pain, Nausea	Granulocytic hyperplasia, Contrast-enhancing mass on CT, Neutrophilia	Percutaneous transhepatic cholangiodrainage	54 days	8
11	Takehiko Takeda, 1990 [34]	79/F	Japan	AD	Pleomorphic giant cell carcinoma	M	Anorexia, fatigue	Elevated ESR ^k^, elevated CRP, hypercalcemia, elevated ALP, dilatation of the duodenal bulb and smooth stenosis of the proximal second part of the duodenum	Cholecystectomy with NIL radical removal of metastatic lesions	8 months	8
12	Kazuhiro Suzumura, 2014 [35]	78/M	Japan	ATD	Adenosquamous carcinoma	LA	RUQ pain, fever	Neutrophilia, elevated CRP, transaminitis, elevated tumour markers	Cholecystectomy with central bisegmentectomy of the liver, lymph node dissection and right hemicolectomy	Alive	7
13	Tetsuo Ikeda, 2005 [36]	50/F	Japan	ATD	Adenocarcinoma	M	Right hypochondrium pain	Hypercellular marrow with neutrophilia Transaminitis Hyperbilirubinemia	Cholecystectomy with extended right lobectomy of the liver Lymph node dissection	Alive	7
14	BM Nagpal VSM, 1998 [37]	50/F	India	ATD	Adenocarcinoma	LA	Painful lump on right side of abdomen, Low grade fever, LOW, Blood in stools	Low Hb count Pulmonary Kochs detected on Chest X-ray Hypotension	Radical Cholecystectomy with en-block removal of the adherent colonic loop and the adjoining liver was carried out	Alive	8
15	Masahiro Yanagi, 2023 [38]	71/M	Japan	ATD	Adenosquamous carcinoma	M	Right sided abdominal pain	Transaminitis, Elevated IL-6, Elevated tumour markers, High PTHrP	GEM ^l^ chemotherapy	27 days	8

^a^ ATD: at the time of diagnosis of GBC, ^b^ AD: after diagnosis of GBC, ^c^ LA: Locally advanced, ^d^ M: Metastatic, ^e^ CRP: C-reactive protein, ^f^ ALT: alanine transaminase, ^g^ ALP: alkaline phosphatase, ^h^ AFP: alpha fetoprotein, ^i^ LDH: lactate dehydrogenase, ^j^ GTP: gamma-glutamyl transpeptidase, ^k^ ESR: erythrocyte sedimentation rate, ^l^ GEM: gemcitabine.

##### Investigations and Management

GBC diagnosis typically follows a two-step process: abdominal ultrasound or contrast-enhanced CT scan [39,40,41]. Liver function tests and inflammatory markers will provide additional clues, though jaundice is a late sign, and inflammatory markers are noted to be inconsistently elevated. Approximately two-thirds of cases showed transaminitis, and one-third had inflammatory marker elevation. Tumour markers, such as CA19-9, were rarely elevated. Metastases to the liver and spine were found in nearly half of the patients. Interestingly, it was noted, from our review, that leukocytosis severity did not necessarily correlate with tumour burden, which suggests that there might be underlying factors contributing to leukocytosis that have yet to be discovered. Radical cholecystectomy remains the primary treatment [42], often leading to reduced WBC counts and G-CSF levels. Chemotherapy and immunotherapy show variable success [43,44].

#### 3.1.2. Paraneoplastic Leukemoid Reaction

##### Pathophysiology

Paraneoplastic Leukemoid Reaction (PLR) is an extreme form of leukocytosis (>50,000/µL) in the absence of hematologic malignancy, often misdiagnosed as infection due to its presentation with fever, weight loss, and fatigue [16]. Unlike standard leukocytosis, PLR is driven by excessive tumour-derived cytokine secretion, predominantly granulocyte-colony stimulating factor (G-CSF) and interleukin-6 (IL-6), which promote neutrophil proliferation and a systemic inflammatory state [17]. This pro-inflammatory microenvironment contributes to tumour progression, angiogenesis, and immune evasion. PLR was most commonly seen in aggressive, high-burden malignancies, including lung, renal, and gastrointestinal cancers, with GBC representing a rarer but significant subset.

##### Results

Among the 15 included studies assessing leukocytosis, five studies presented with PLR [16,28,33,34,37].

##### Investigations and Management

Peripheral blood smears in PLR typically revealed granulocytosis without immature blasts, differentiating it from leukaemia. Bone marrow biopsy findings are often normal or show reactive hyperplasia without dysplasia, further distinguishing PLR from primary hematologic disorders. Importantly, PLR is associated with poor prognosis, often correlating with advanced disease, high metastatic burden, and reduced survival. Among the included cases, PLR was exclusively observed in metastatic GBC, reinforcing its role as a marker of tumour aggressiveness. Management of PLR remains largely supportive, with leukapheresis considered in extreme cases. Corticosteroids and NSAIDs have been explored for symptomatic relief, though definitive management requires tumour-directed therapy, which has been shown to reduce leukocyte counts post-treatment [45]. Platinum-based regimens, particularly gemcitabine–cisplatin, have demonstrated efficacy in controlling both tumour progression and cytokine-driven leukocytosis in GBC. However, no standardised approach exists for PLR management, and further research is needed to determine whether it could serve as an early biomarker for aggressive tumour behaviour.

### 3.2. Dermatological Paraneoplastic Syndromes

#### 3.2.1. Inflammatory Myositis—Dermatomyositis and Polymyositis

##### Pathophysiology

**Figure 3:**
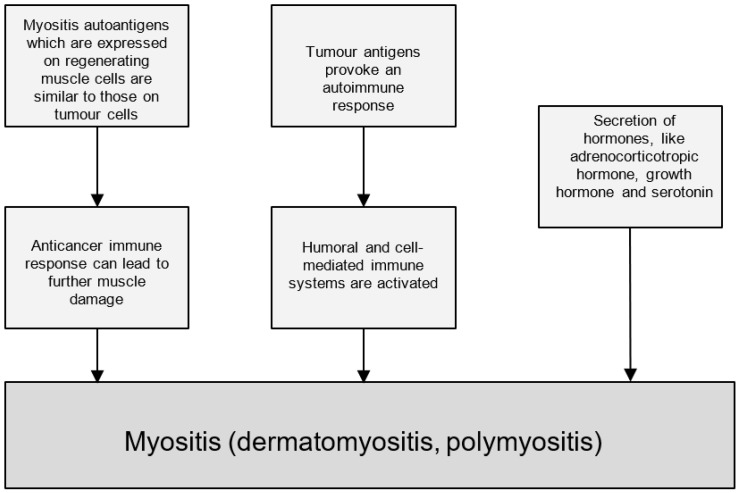
Flowchart showing postulated mechanisms linking paraneoplastic inflammatory myositis to malignancies.

Inflammatory myositis, encompassing dermatomyositis and polymyositis, is an inflammatory myopathy characterised by proximal muscle weakness [46]. The initial association between inflammatory myositis and malignancy was first noted over a century ago [47]; however, it remains a relatively uncommon occurrence, affecting a significant minority of cancer patients. While paraneoplastic processes are understood to be linked to oncogenesis and autoimmunity, the precise pathophysiological mechanisms underlying paraneoplastic inflammatory myositis remain elusive [46]. The detection of myositis-specific antibodies provides evidence for this association [48]. Casciola-Rosen et al. proposed a mechanism where myositis autoantigens, expressed in regenerating muscle cells, share similarities with those found on tumour cells [49]. This suggests that an anticancer immune response targeting tumour cells could inadvertently cause muscle damage. Other proposed mechanisms include the involvement of humoral and cell-mediated immune responses and the presence of tumour antigens eliciting an autoimmune reaction [50]. Additionally, the secretion of hormones such as adrenocorticotropic hormone, growth hormone, and serotonin has been implicated as a potential pathogenic factor [51]. A schematic representation of these proposed pathophysiological mechanisms is depicted in Figure 3.

Dermatomyositis presents characteristically with symmetrical proximal muscle weakness and a variety of dermatological manifestations, including a heliotrope rash and Gottron’s papules [51]. Other associated features may include eyelid oedema, pitting oedema, dysphagia, nasal regurgitation or aspiration pneumonia, and dyspnea. Notably, dermatological findings may be the sole manifestation in up to 40% of patients [51]. The pathophysiology of dermatomyositis classically involves the binding of immune complexes to endothelial cells. This triggers the complement system via the membrane-attack complex, leading to cell lysis and endothelial cell necrosis. The resulting impaired blood supply likely contributes to perifascicular atrophy. However, recent research suggests that a type I interferon-mediated cascade may be the predominant underlying mechanism [52].

Polymyositis presents with proximal muscle weakness, usually most severely in the pelvic girdle and shoulders [53], and commonly in the neck flexors [54]. This is accompanied by a marked elevation of creatinine kinase. Its pathogenesis is characterised by local activation of immune cells in skeletal muscle. This is due to the expression of cytokines such as IFN-γ, IL-6, IL-1β, TNF-α, and TGF-β [55] and chemokines like IL-8, CCL-2, CCL-3, CCL-4, CCL-5, CXCL-9, and CXCL-10 [56].

##### Results: Dermatomyositis

This review analysed nine case reports, encompassing seven females and two males with a mean age of 67.7 years (range: 48–90 years), who presented with dermatomyositis as the primary PNS. Table 2 summarises the timing of dermatomyositis diagnosis in relation to GBC, clinical presentation, investigations performed, management, and survival outcome.

##### Investigations and Management: Dermatomyositis

In all patients with dermatomyositis, the diagnosis was made either before or concurrently with the diagnosis of GBC, likely due to the characteristic features of dermatomyositis being the presenting symptoms. The majority of patients (88.9%) presented with a facial and truncal rash, often accompanied by proximal muscle weakness. Late-stage symptoms such as dysphagia were also observed in over half of the patients (55.6%). All cases of dermatomyositis as a PNS arose in the setting of locally advanced or metastatic GBC. Extensive investigations were conducted, revealing elevated creatinine kinase levels in most patients. Serum autoantibody testing showed that 75% of patients were positive for ANA. Tumour markers were less reliable, with some patients exhibiting elevated CA-125 and carcinoembryonic antigen levels, while others had normal values. To confirm the diagnosis of dermatomyositis, electromyography, muscle biopsy, and skin biopsy were performed in some cases, with findings consistent with the diagnosis. However, in most cases, the characteristic clinical features, including dermatological manifestations and proximal muscle weakness, were sufficient for a clinical diagnosis of dermatomyositis.

Management of dermatomyositis in the context of GBC generally involves three components: immunosuppression, anticancer therapy, and surgery. Corticosteroids are the mainstay of dermatomyositis treatment and are typically administered until clinical improvement in both dermatological manifestations and muscle weakness is observed. The dosage is then tapered gradually. Other immunosuppressive agents, such as methotrexate and hydroxychloroquine, remain adjuncts. In this cohort, 77.8% of patients received immunosuppression, with methylprednisolone as the most common agent. Outcomes were positive, with 85.7% of patients showing improvement in muscle strength and/or regression of dermatological manifestations. Only one patient experienced regression of skin lesions but with concurrent progression of muscle weakness. Alternative approaches to managing dermatomyositis beyond corticosteroids have also been explored. Ni Q. F. et al. [51] reported promising results using a combination therapy consisting of loratadine, cetirizine, hydroxychloroquine, magnesium isoglycyrrhizinate, vitamin C, calcium gluconate, and a coenzyme complex, resulting in gradual abatement of skin lesions [51]. Of note, there is some evidence [57,58] describing an improvement in dermatomyositis after chemotherapy, which suggests that the immunosuppressive effect of systemic chemotherapy contributes to the improvement of dermatomyositis. Anticancer treatment, in the form of chemotherapy or chemoradiotherapy, was administered to 33.3% of patients. However, many patients were ineligible for such treatments due to advanced GBC stage or advanced age. Gemcitabine and cisplatin were the most common chemotherapeutic agents used. While the specific impact of anticancer treatment could not be definitively determined, all three patients who received it ultimately passed away. Surgical intervention was performed in 44.4% of patients. Patients who underwent radical cholecystectomy exhibited more favourable outcomes compared to those who underwent exploratory laparotomy. This is aligned with the current literature indicating that exploratory laparotomy often reveals previously undetected peritoneal and liver metastases, leading to a poorer prognosis.

**Table 2:** Studies reporting paraneoplastic dermatomyositis associated with gallbladder cancer (GBC).

S/N	First Author, Year	Age/Sex	Country	Timing of PNS Diagnosis	Type of Cancer	Tumour Characteristic	Presentation	Investigations	Management	Survival Time After PNS Diagnosis	Study Bias
1	Jurcic P, 2015 [50]	48/F	Croatia	ATD	Adenocarcinoma	M	Face and neck erythema Butterfly-shaped rash Pain in upper arm muscles Stiffness of hands Dysphagia	High ESR ^k^ and CK Weakly positive ANA	Exploratory laparotomy Chemotherapy (cisplatin, gemcitabine) Immunosuppression	Not stated Palliation	8
2	Ni Q. F, 2013 [51]	67/F	China	ATD	Adenocarcinoma	LA	Facial and cervical erythema Pruritus Arthralgia of hands Morning stiffness	High CRP Positive skin biopsy findings	Symptomatic treatment Radical cholecystectomy	Discharged on POD ^p^ 14 Alive 5 months after first presentation	7
3	Aritra Paul, 2020 [52]	64/M	India	ATD ^b^	Signet ring cell carcinoma	M	Cervical lymphadenopathy Facial rashes Periorbital oedema Nasal regurgitation Dysphagia Weakness in limbs	-	-	-	4
4	D. A. Narasimhaiah, 2011 [59]	65/F	India	BD ^a^	Adenocarcinoma	M ^c^	Erythematous rash (face and upper limbs) Progressive proximal weakness Dysphagia Nasal regurgitation	High CK ^e^, LDH ^f^, CEA ^g^ and CA-125 Weakly positive ANA h Positive muscle biopsy findings	Immunosuppression	Not stated Patient demise due to DVT ^i^ and subsequent PE ^j^, and aspiration pneumonia	8
5	Park J. S, 2012 [60]	71/M	Korea	ATD	Not stated	M	Heliotrope rash Gottron’s papules Shawl sign Itchy rash on face and trunk Myalgia and muscle weakness	High ESR and CRP ^l^ Positive RF ^m^ and ANA Positive muscle and skin biopsy findings	Immunosuppression Chemoradiotherapy (5-fluorouracil)	Passed away due to acute renal failure from peritoneal carcinomatosis	8
6	Garg T, 2017 [61]	55/F	India	BD	Adenocarcinoma	LA ^d^	Generalised oedema Erythema on face, neck, chest, shoulders, thighs and buttocks Proximal muscle weakness	High ESR and CRP Positive ANA and anti-dsDNA ^n^ Positive muscle and skin biopsy findings	Immunosuppression Extended cholecystectomy	Alive	6
7	Takahiro Sawada, 2014 [62]	90/F	Japan	ATD	Not stated	-	Heliotrope rash Erythema keratodes Gottron’s papules Muscle weakness	High CK Positive ANA	Non operative management due to advanced GBC and old age Muscle functional rehabilitation	Not stated Palliation	8
8	Kuroda H, 2022 [63]	75/F	Japan	ATD	Adenocarcinoma	M	General fatigue Muscle weakness Dysphagia Generalised erythema	High CK, CRP, and LDH Positive TIF1-γ ^o^ Positive muscle and skin biopsy findings	Immunosuppression Chemotherapy (cisplatin, gemcitabine)	Passed away due to pneumonia	8
9	Yiannopoulos G, 2002 [64]	75/F	Greece	BD	Adenocarcinoma	M	Proximal muscle weakness Dysphagia Dysphonia Facial erythema Oedema of eyelids	Elevated LDH Positive ANA Positive muscle biopsy findings	Immunosuppression Exploratory laparotomy (2 months after initial presentation)	Passed away 10 weeks after laparotomy due to pneumonia	8

^a^ BD: Before diagnosis of GBC, ^b^ ATD: At the time of diagnosis of GBC, ^c^ M: Metastatic, ^d^ LA: Locally advanced, ^e^ CK: Creatinine kinase, ^f^ LDH: Lactate dehydrogenase, ^g^ CEA: Carcinoembryonic antigen, ^h^ ANA: Antinuclear antibody, ^i^ DVT: Deep vein thrombosis, ^j^ PE: Pulmonary embolism, ^k^ ESR: Erythrocyte sedimentation rate, ^l^ CRP: C-reactive protein, ^m^ RF: Rheumatoid factor, ^n^ anti-dsDNA: Anti-double stranded deoxyribonucleic acid, ^o^ TIF1-γ: Transcription intermediary factor 1 gamma, ^p^ POD: post-operative day.

##### Results: Polymyositis

A case report described a 68-year-old woman with pain and muscle weakness who, despite normal immune markers like ANA, was diagnosed with polymyositis and concurrent GBC [60].

##### Investigations and Management: Polymyositis

She was treated with corticosteroids (prednisolone) for polymyositis and underwent an extended cholecystectomy. Eight months after surgery, she was reportedly doing well with no signs of cancer recurrence or polymyositis.

#### 3.2.2. Acanthosis Nigricans and Sign of Leser–Trélat

##### Pathophysiology

Acanthosis nigricans (AN) is most commonly associated with diabetes and insulin resistance, but it can also be a rare sign of internal malignancy [56], most frequently gastric, pancreatic, and lung cancers [65]. This distinction allows for its classification into malignant and non-malignant cases. While non-malignant AN can occur at any age [66], malignant AN primarily affects middle-aged and elderly patients. The precise mechanism underlying AN in the context of internal malignancy is not fully understood. Some theories propose that malignant tumours release growth-promoting factors, such as platelet-derived growth factor and epidermal growth factor [67], or that they secrete hormones stimulating melanocyte-stimulating hormones, leading to increased growth of melanocytes, fibroblasts, and keratinocytes [68]. Another hypothesis suggests that malignant AN is caused by the ectopic release of a peptide from the neoplasm [69]. AN is characterised by hyperpigmentation of the skin with a velvet-like texture. It most commonly affects flexural areas and skin folds, such as the neck, axilla, and knuckles, displaying a symmetrical distribution [70]. In malignant AN, skin thickening and characteristic skin lesions may be observed, potentially at the junction of mucous membrane and skin mucosa. Patients may also experience itching or irritation.

Leser–Trélat syndrome is a rare cutaneous paraneoplastic phenomenon often associated with internal malignancies. This syndrome is characterised by the sudden appearance and rapid increase in size and number of seborrheic keratoses (warty growths) on the thorax and dorsum, accompanied by pruritus [71]. While its pathophysiology is not fully understood, factors like human growth factor, Epidermal Growth Factor alpha, and the Epidermal Growth Factor Receptor are believed to contribute to seborrheic keratosis development [72]. AN is frequently observed in conjunction with Leser–Trélat syndrome. It has been suggested that the growth factors responsible for seborrheic keratosis may also affect flexures, resulting in hyperpigmentation and papillomatosis, characteristics typical of AN [73].

##### Results

This review extracted three case reports, consisting of three females with a mean age of 52 years (range: 45–56 years). As shown in Table 3, the majority presented with characteristic skin lesions—hyperpigmentation of flexural areas and skin folds. Notably, one patient also presented concurrently with Leser–Trélat syndrome. 

##### Investigations and Management

AN is typically diagnosed clinically and confirmed with a skin biopsy. In these patients, the diagnosis of AN was made clinically either before or at the time of diagnosis of GBC. Treatment of the underlying malignancy, GBC in this case, often leads to regression of AN [73]. Hence, management primarily consisted of anticancer treatment in the form of chemotherapy.

#### 3.2.3. Sweet’s Syndrome

##### Pathophysiology

Sweet’s syndrome [75], also called acute febrile neutrophilic dermatosis, is a rare disorder that often presents with acute tender plaques or nodules and other systemic symptoms such as fever, arthralgia, and ophthalmological manifestations. Sweet’s syndrome usually occurs without an identifiable cause but is often associated with other conditions. These conditions include infections, autoimmune diseases, and cancers. While it is most frequently related to blood cancers, like acute myeloid leukaemia, Sweet’s syndrome can also be connected to solid tumours, especially genitourinary cancers [76,77].

##### Results

There is only one reported case of Sweet’s syndrome manifesting as a GBC PNS [77]—a 45-year-old woman who presented with painful, red blisters (bullae).

##### Investigations and Management

The patient was diagnosed with GBC through an abdominal ultrasound. The presence of these blisters, along with non-specific symptoms like fever and malaise, pointed towards Sweet’s syndrome, allowing for an early diagnosis of GBC. The exact cause of Sweet’s syndrome is unknown, but it is believed to involve an abnormal immune response, possibly a hypersensitivity reaction. The patient was successfully treated with open cholecystectomy and corticosteroids. Diagnostic criteria [78] for Sweet’s syndrome were first proposed by Su and Liu and further modified by von den Dreisch. Diagnostic criteria for Sweet’s syndrome are divided into major and minor criteria. Major criteria include the following: (a) abrupt onset of tender erythematous plaques or nodules with vesicles, pustules, or bullae; (b) predominant neutrophilic infiltration in the dermis without leukocytoclastic vasculitis. Minor criteria include the following: (a) eruption is preceded by a non-specific respiratory/gastrointestinal infection, inflammatory/autoimmune disorder, neoplasia, or pregnancy; (b) periods of general malaise and fever (>38 °C); (c) raised ESR > 20 mm, raised CRP, and leukocytosis; (d) excellent response to treatment with systemic corticosteroids or potassium iodide. A diagnosis of Sweet’s syndrome can be made upon fulfilment of both major criteria and two minor criteria. The syndrome is typically treated with topical or intralesional corticosteroids, but, if those are ineffective, other medications such as colchicine or potassium iodide can be considered.

#### 3.2.4. Exfoliative Dermatitis (Erythroderma)

##### Pathophysiology

Exfoliative dermatitis, or erythroderma, is a rare presentation associated with GBC. While the exact pathogenesis of erythroderma is unknown, some have postulated that it is due to increased expression of adhesion molecules in epithelial cells, leading to increased dermal inflammation and epidermal proliferation [79]. This characteristically manifests as diffuse erythema and scaling of greater than 90% of the body surface area [80].

##### Results

In the two reviewed case studies, patients were diagnosed with GBC concurrently with the development of exfoliative dermatitis, often characterised by widespread erythematous, pruritic rashes. The first case involved a 77-year-old man whose initial symptoms included extensive skin rash, leading to the discovery of asymptomatic GBC [81]. The next case described a 71-year-old male who presented with an exfoliative skin rash and was ultimately found to have a large gallbladder mass diagnosed as adenocarcinoma [82].

##### Investigations and Management

For the first case, following surgical intervention, including an extended cholecystectomy, the patient experienced significant improvement in skin lesions within one-week post-operation, suggesting that addressing the underlying malignancy can result in the regression of associated dermatological conditions. For the second case, the symptom of exfoliative dermatitis served as a critical clinical warning sign, facilitating the early diagnosis and timely treatment of GBC. Both cases underline the importance of recognising exfoliative dermatitis as a potential PNS in GBC patients.

### 3.3. Neurological Paraneoplastic Syndromes

#### 3.3.1. Pathophysiology

Paraneoplastic neurological and neuromuscular syndromes can occur in many types of cancer. In blood cancers, such as leukaemia and paraproteinemia, various nerve-related conditions have been documented, including paraneoplastic sensorimotor and sensory neuropathy, sensory neuronopathy, motor and autonomic nerve issues, cranial nerve problems, and demyelinating and vasculitic neuropathies [83,84]. In solid organ tumours, such as hepatocellular carcinoma, Guillain Barre syndrome, chronic inflammatory demyelinating polyneuropathy, anti-GMI ganglioside antibody-positive degenerative axonal polyneuropathy, anti-Hu antibody-positive peripheral neuropathy, and PR3-Antineutrophil-Cytoplasmic-Antibody-positive demyelinating polyneuropathy are the possible subtypes of paraneoplastic neuropathy [85,86,87,88,89].

#### 3.3.2. Results

In GBC, neurological and neuromuscular conditions can present as PNS. Perhaps the most significant of them would be that of paraneoplastic peripheral neuropathy, for which Guillain Barre syndrome and anti-Hu-associated paraneoplastic sensorimotor neuropathy have been reported [90,91,92,93]. Other paraneoplastic neurological presentations include Lambert–Eaton myasthenic syndrome, paraneoplastic cerebellar degeneration, multiple cranial nerve palsies, and even the neuromuscular condition of opsoclonus–myoclonus syndrome [94,95,96,97,98]. This paper extracted nine cases of the aforementioned neurological PNS in GBC patients.

In the cases of peripheral neuropathy, patients may present progressive lower limb paraesthesia and weakness affecting their gait and ambulance [94,96,97]. In addition, patients with paraneoplastic Guillain-Barre syndrome may also experience facial diplegia, bulbar palsy, and flaccid paralysis in all four limbs at a later stage of the disease [99]. In comparison, other neurological PNS tend to have more wide-ranging and bizarre presentations, such as profound vertigo [95,97,98], diplopia [94,96,97], proximal weakness worsening at the end of the day [94], gait and limb ataxia alongside other cerebellar signs [95], ptosis [96], hyperhidrosis [94], and mental obtundation [97,98].

#### 3.3.3. Investigations and Management

Nerve conduction studies and Western immunoblotting have been employed to aid with the diagnostic process. Serological testing and cerebrospinal fluid analysis may prove to be useful. Neuroimaging may also be useful to rule out any confounding factors, like brain lesions [95,97,98]. These neurologic symptoms respond partially to chemotherapy with agents like cisplatin, as well as cycles of plasma exchanges. In the case of opsoclonus–myoclonus syndrome, intravenous immunoglobulin and IV corticosteroids may be used for treatment; however, there are inconsistent outcomes of treatment with complete resolution in Brandes et al.’s 2020 study as compared to minimal to no effect with the patient eventually succumbing to the malignancy in 5 weeks in Corcia et al.’s study more than two decades earlier [96,98]. On the other hand, Kaido et al.’s study has shown that complete resolution of the symptoms and the underlying malignancy is possible with surgical extirpation of the cancer itself followed by adjuvant chemotherapy, with the patient being alive after 5 years despite failure of the initial treatment of steroid pulse therapy targeted towards the symptoms of cranial nerve palsy [97]. This study also alluded to the importance of understanding that neurological symptoms not responding to conventional therapy could be an atypical early presentation of an otherwise asymptomatic underlying malignancy (in this case GBC), and with this understanding early detection and complete eradication of cancer is possible.

### 3.4. Metabolic Paraneoplastic Syndromes

#### 3.4.1. Hypercalcaemia

##### Pathophysiology

Hypercalcaemia, a common PNS, occurs in 20–30% of all cancer cases [99]. Calcium is the most abundant mineral in the body and is crucial for various functions. It is essential for bone health and growth, neuromuscular activity, and digestive health, and functions as a vital secondary messenger in many cell signalling pathways [100]. Because calcium is vital for many essential body functions, it must be tightly regulated through the body’s homeostasis mechanisms, such as hormonal control by the parathyroid hormone [101] This keeps the serum calcium in a narrow range from 8.5 to 10.5 mg/dl (from 4.3 to 5.3 mEq/L or from 2.2 to 2.7 mmol/L) (some calcium is also bound in albumin in human serum, hence calculating the corrected calcium levels is crucial to account for the albumin-bound calcium) [102]. If calcium levels in the body go outside the normal range it can lead to a wide range of serious health problems. High calcium levels, or hypercalcaemia, can cause kidney stones, bone disorders, digestive issues, and psychiatric symptoms, often summarised as “stones, bones, groans, and moans”. These effects can be particularly severe for cancer patients experiencing the related PNS [103].

Paraneoplastic hypercalcaemia can present in a multitude of cancers, including hepatocellular carcinoma, ovarian, renal, thyroid, melanoma, lung cancer, haematological malignancies, and other neuroendocrine tumours [104]. Presentation of hypercalcaemia in a GBC patient is rare, given the rarity of the primary cancer [105]. Hypercalcaemia in a patient with primary GBC was first described by M Takahashi et al. in 1985 [34]; since then, at least eight other cases of such paraneoplastic hypercalcaemia have been reported.

**Figure 4:**
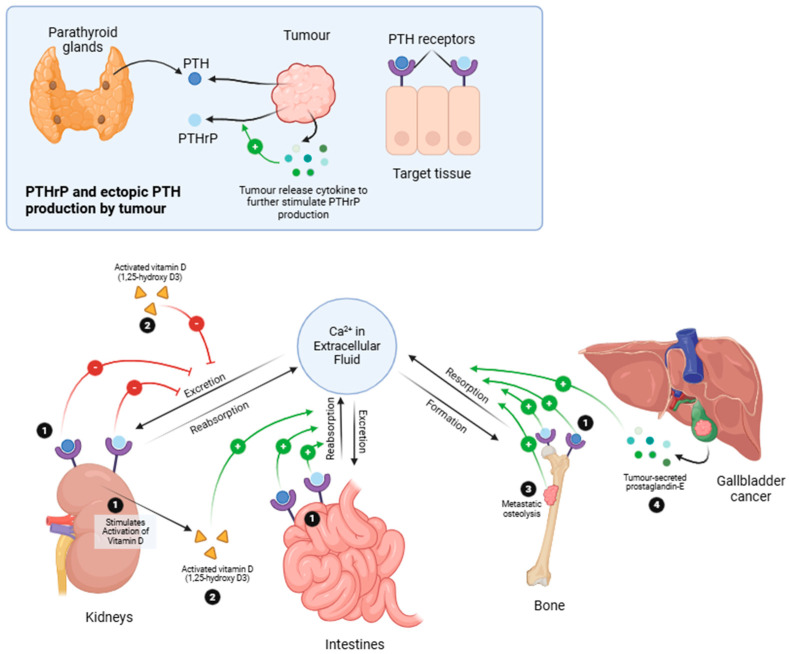
Proposed pathophysiology of paraneoplastic hypercalcaemia.

Paraneoplastic hypercalcaemia can arise through various mechanisms of malignancy, as summarised in Figure 4. The most common cause typically involves tumours producing parathyroid hormone-related peptide (PTHrP), which shares structural similarities with PTH (parathyroid hormone) and interacts with the same receptor (PTH receptor) in target tissues to increase serum calcium levels [106]. Fuller Albright initially identified humoral hypercalcaemia of malignancy, suggesting its association with either parathyroid hormone (PTH) or a comparable peptide [107]. The discovery of parathyroid hormone-related protein (PTHrP) linked it to the onset of humoral hypercalcaemia in malignancies. Hence, the authors investigated serum intact PTH levels in gallbladder patients to dichotomise PTH-dependent hypercalcaemia and PTH-independent mechanisms. When there are elevated levels of PTH, the next step would be to conduct thyroid imaging and Sestamibi scans to rule out parathyroid tumours [34]. If initial neck Sestamibi scans fail to identify the source of PTH production, it is recommended to follow up with a scan of the thorax and abdomen. If PTH levels are normal, serum levels of PTHrP and vitamin D should be checked. Further evidence may include low serum phosphate, hinting at a PTH or PTHrP-related issue, while high serum alkaline phosphatase levels could suggest increased bone resorption or osteolytic lesions. Bone imaging and a myeloma screen can also aid in excluding osteolytic bone lesions [34].

Other less common causes include osteolysis, ectopic production of PTH by tumours, excess production of extra-renal calcitriol, and mechanisms mediated by prostaglandins [39]. In the context of primary GBC, M Takahashi et al. suggested that tumour-secreted PTHrP might induce hypercalcaemia [34], a proposition later confirmed by Y Watanabe et al. and M Yanagi et al., whereby serum PTHrP levels in both patients with primary gallbladder in the background of normal PTH and vitamin D levels [11,39]. However, the rate of PTHrP secretion by the tumour and its biological potency can influence the occurrence and severity of hypercalcaemia [108]. Interestingly, patients with these PTHrp-producing tumours also displayed neutrophilia, which could be attributed to the colony-stimulating factor secreted by the tumour [39,109]. Additionally, M Yogarajah et al. identified the ectopic production of intact PTH by the tumour as another pathway leading to hypercalcaemia in GBC patients [110].

##### Results

Table 4 compiles the findings of five studies (five patients) which reported hypercalcaemia in GBC. Common symptoms include lethargy, generalised weakness, poor feeding, and abdominal pain. In more severe cases, the patient may also present with arrhythmias and an altered mental state. Fever is also noted in patients with concurrent neutrophilia in CSF-producing gallbladder tumours. Of the five patients with paraneoplastic hypercalcaemia, the PNS was the first presentation of the diagnosis in three patients, while it was discovered after the diagnosis for the other two.

Notably, K Takahashi et al. demonstrated, in their 2024 study, that the pathophysiological role that PTHrP plays in humoral hypercalcaemia of malignancy is not limited to increasing the calcium levels but also to the broader phenomenon of cancer cachexia [108]. This was previously elucidated by Kir et al., who discovered that PTHrP’s involvement in thermogenic gene activation within adipose tissues is significant for wasting [111]. Moreover, humoral hypercalcaemia of malignancy significantly impacts the prognosis of cancer patients, with survival typically ranging from one to three months after the onset of hypercalcaemia [11,102]. In advanced malignant tumour cases where hypercalcaemia and elevated blood PTHrP levels coincide, high blood PTHrP concentrations are associated with poorer prognosis, particularly in patients under 65 years old [99].

##### Investigations and Management

Most cases of severe hypercalcaemia are treated with hydration with intravenous fluids, calcitonin, and bisphosphonate therapy to lower serum calcium [39,108,110]. Intravenous pamidronate is recommended for hypercalcaemia of malignancy [110]. From the six cases of GBC, however, the conventional treatment of intravenous hydration, calcitonin, furosemide, and pamidronate either has a minimal response or has a temporary response with subsequent relapse. On the other hand, less orthodox treatment with cinacalcet and mithramycin yields resolution of the hypercalcaemia [11,110]. Source control of PTHrP, such as surgical guidance chemoembolisation with OK432 immunopotentiator and gemcitabine chemotherapy, yields a heterogeneous response in controlling hypercalcaemia, possibly due to the late-stage presentation of GBC with the presence of organ metastasis [34,39]. However, more evidence is needed for the survival benefits of resolution of paraneoplastic hypercalcaemia in GBC patients.

#### 3.4.2. Hyponatraemia

##### Pathophysiology

Hyponatraemia is another GBC PNS with important metabolic implications [112]. Most commonly occurring in lung cancer (particularly small-cell lung carcinoma), hyponatraemia can also occur in a plethora of malignancies, including cancers of the prostate, pancreas, liver, and kidney, and is a known negative prognostic marker [113,114]. Hyponatraemia as a PNS was first illustrated by Ng Esther et al. in 2010 in a 35-year-old woman with metastatic GBC [115].

Sodium is a major cation in the extracellular fluid in the human body [116]. Sodium plays a vital role in maintaining cellular homeostasis, regulating fluid and electrolyte balance, blood pressure, muscle and nerve cell excitability, and facilitating nutrient transport through plasma membranes [117]. Due to its importance in influencing the osmolality of the human body in addition to its metabolic significance, sodium is kept in a strict concentration range of 135–145 mmol/L and is regulated tightly by the renal and endocrine system [117]. When the regulation fails, hyponatraemia occurs and this causes haemodynamic imbalances, neurologic dysfunction, altered cognition, cerebral oedema, gait disturbances and falls, osteoporosis, and fractures [118], thereby causing increasing morbidity and mortality in cancer patients [119].

There is a wide range of causes for hyponatremia in a cancer patient, ranging from factitious causes due to severe hyperproteinemia, hyperlipidemia, hyperglycemia, gastrointestinal fluid loss, and related causes, such as kidney, adrenal, liver failure and hypothyroidism, and even primary polydipsia [120,121]. Paraneoplastic causes of hyponatraemia, however, classically arises from the ectopic production of arginine vasopressin of the tumour—commonly described as the Syndrome of Inappropriate Antidiuretic Hormone production (SIADH) [122,123]. The ectopic production and secretion of arginine vasopressin were proven in small-cell lung cancer-associated hyponatremia [124,125,126]. Specifically, for GBC, studies by Ng Esther et al. [115] and Saleem et al. [127] postulated SIADH as a possible mechanism for paraneoplastic hyponatremia. This is confirmed by Tamura et al., where raised ADH levels of 5.8 pg/mL (normal: 0.3–3.5 pg/mL) in the background of normal CT thorax and CT/MRI brain (no pulmonary or pituitary lesions) and normal thyroid function test and adrenal function tests, elucidating the mechanism of SIADH from ectopic production by small-cell GBC [9]. Researchers have explored the role of atrial natriuretic peptide in paraneoplastic hyponatremia associated with cancer, particularly its potential involvement beyond the typical role of arginine vasopressin. However, this mechanism has not been specifically studied in GBC PNS [121,128,129].

##### Results

Table 5 summarises four cases of GBC associated with paraneoplastic hyponatremia. While epigastric pain and jaundice are symptoms indicating primary GBC and liver metastasis, paraneoplastic hyponatraemia presents with non-specific symptoms, such as general fatigue, weakness, anorexia, nausea, and vomiting, which can appear early in the disease. 

##### Investigations and Management

As the abovementioned symptoms might result from other factors, comprehensive blood tests, serum biochemistry, imaging of the lungs and brain, and thyroid and adrenal function tests are conducted to rule out other causes. This approach is illustrated in the 2022 study by Gorrepati et al., where normal serum osmolality despite low sodium levels led to further tests revealing hyperglycemia and hyperuricemia, ultimately resulting in the diagnosis of pseudohyponatremia [127]. Moreover, hyponatraemia may be the first presenting symptom that heralds the diagnosis of GBC. In two out of four cases, hyponatraemia is the first sign of GBC. Thus, a physical exam is imperative in prompting gallbladder imaging modalities, like ultrasound and endoscopic retrograde cholangiopancreatography (ERCP), as well as biopsy and cytological testing to confirm the diagnosis. Such early presentation of symptoms suggesting hyponatraemia also underscores the importance of hyponatraemia in aiding with the early detection of a primary malignancy, which could potentially improve treatment and patient outcomes [114].

Conventional treatments for SIADH in cancer would mirror those used for hyponatremia—fluid restriction, increased intake of osmotic solutes to enhance clearance of water, or even the use of selective vasopressin receptor antagonists [114]. Infusion of hypertonic saline could also be employed for patients with more symptomatic hyponatremia [114]. Nevertheless, for the three GBC patients with true paraneoplastic hyponatremia, the conventional standard of care results in partial resolution at best for one patient and minimal to no response or even negative response for two other patients [8,115,127]. The patient with pseudohyponatremia was started on cholestyramine and ursodiol and was discharged 2 weeks after the normalising of serum sodium levels [127]. Combined with source control via systemic chemotherapy (cisplatin and etoposide) or resection of the metastatic nodule, the treatment outcomes improved further, but a full resolution was still unable to be achieved. The one case with eventual optimal control of hyponatraemia involves the use of mozavaptan, even with the loosening of water restrictions [127]. While more evidence is required, this case sheds light on how vaptans could be the key to controlling paraneoplastic SIADH in GBC and improving patients’ quality of life.

### 3.5. Other Paraneoplastic Syndromes

#### 3.5.1. Chorea

##### Pathophysiology

Another unique PNS reported in the literature is chorea. Chorea is an extremely rare PNS, with the majority of cases linked to small-cell lung cancer, with the exact mechanism of paraneoplastic chorea being not well-established. Previous studies [131,132] have suggested that the manifestation of chorea in cancer has a strong link with CV2/CRMP5 antibodies. Not enough is known about it to postulate its pathophysiology. It is more commonly linked to Huntington’s disease, which is normally attributed to the expansion of cytosine–adenine–guanine (CAG) repeats in the huntingtin gene [133]. Chorea as a PNS is more commonly associated with solid tumours such as small-cell lung carcinoma and thymomas [134]. The characteristic choreic movements normally develop as part of a more extensive involvement of the nervous system that may include other neurological conditions, such as cerebellar ataxia, peripheral neuropathy, or optic neuritis [135,136,137].

##### Results

To date, there is only one known case of chorea associated with primary GBC [138]. The patient is an 81-year-old female who presented with involuntary movement of her left extremities.

##### Investigations and Management

She was diagnosed with GBC via a contrast CT scan and was managed successfully with a gemcitabine–cisplatin chemotherapy protocol. Interestingly, the patient did not test positive for any serum antibodies and administering haloperidol was found to be ineffective. However, due to the circumstances and nature of the symptoms, the patient was still diagnosed with paraneoplastic chorea, which was proven to be accurate as symptoms resolved upon administering treatment. Paraneoplastic chorea is very hard to detect, mostly showing no abnormalities in scans such as magnetic resonance imaging (MRI) scans and CTs and only manifesting clinically [139]. Diagnosis of chorea is often made clinically, as well as by testing for high-risk antibodies such as CRMP5 and ANNA1 IgG [140]. It typically does not have a good prognosis, being resistant to several medications, such as neuroleptics, benzodiazepines, anticonvulsants, and corticosteroids [141].

### 3.6. Combined Paraneoplastic Syndromes

#### Haematological with Concurrent Metabolic Syndrome

An interesting outcome to note was the presence of patients with concurrent multiple paraneoplastic syndromes diagnosed at the same time. M.Yanagi et al. [38], T.Takeda et al. [34], Y.Watanabe et al. [11], and M.Takahashi et al. [33] published studies of patients presenting with paraneoplastic syndromes of both leukocytosis and hypercalcaemia concurrently. There is not much similarity between the patients in these studies other than the fact that all the patients presented with their symptoms in Japan. Hypercalcaemia and leukocytosis by themselves are considered fairly common among the rare subgroup of paraneoplastic syndromes and have been extensively proven to arise from tumour cells and not other pathologies happening at the same time [142]. Hypercalcaemia and leukocytosis together, however, is a rare occurrence with much of its current mechanism unknown. O. Burzyantseva et al. [143] hypothesised that the secretion of haematopoietic factors, such as G-CSF, PTHrP, IL-1, etc., would trigger the release of leukocytes that subsequently act on the common precursors of osteoclasts and granulomonocytic cells to cause hypercalcaemia. A few other cases [144,145] of paraneoplastic leukocytosis–hypercalcaemia were reported, but the cancers in these cases were mostly of the lung and not the gallbladder. Finally, a retrospective cohort study by A.Hiraki [146] showed that paraneoplastic leukocytosis–hypercalcaemia is highly associated with higher mortality in cancer patients compared to just any of the pareneoplastic syndromes alone. Thus, the above studies all stress the importance of conducting calcium assays in cancer patients who present with leukocytosis to check for concurrent syndromes. Early recognition of the syndrome followed by surgical excision of the tumour as the definitive treatment is crucial in lowering the mortality of such patients.

## 4. Strengths

This review highlighted the numerous PNS associated with gallbladder cancers. To our knowledge, this study is the first of its kind to highlight more than just the different PNSs but also to show the relevant laboratory findings that can help with earlier identification of these syndromes and the management strategies adopted by various clinicians to address them. Although further larger studies are needed to establish a risk ratio between gallbladder cancer and these syndromes, the underlying treatment implications are significant enough that they warrant future clinicians looking out for and addressing these PNSs when treating patients with gallbladder cancer. Despite many of the cases in our review being from Asian countries, gallbladder cancer was also found to have a high prevalence in Western countries, such as the USA. The results of this paper are thus likely to be transferable and can apply to other ethnic populations as well; further research into this is warranted.

## 5. Limitations

Due to the rarity of this topic, most of the available literature consists of case reports, which restricts any statistical analysis. A key challenge is determining whether symptoms like hyponatraemia or leukocytosis are truly paraneoplastic effects of GBC. Typically, a symptom is considered paraneoplastic if it correlates with cancer onset and is not a standard manifestation of the cancer itself. Such findings are often incidental and rare, making them impractical to actively seek out. Moreover, few existing studies report an association or shared mutations between GBC and other well-characterised cancers. While some genetic mutations found in GBC, like TP53 and KRAS, were also present in other cancers, no published research explores their mechanistic links. Common paraneoplastic syndromes, such as dermatomyositis, are more commonly observed in patients with other common cancers like lung and colon cancers. However, the current literature does not suggest whether a diagnosis of GBC with dermatomyositis justifies further investigation for lung or colon cancer or vice versa, given the majority of GBCs were diagnosed at late stages. Although some symptom improvements after tumour removal suggested a paraneoplastic nature, there is the potential for high bias in case reports due to inadequate follow-up, preventing definitive conclusions. These syndromes often appeared in advanced cancer stages, complicating the analysis of treatment impacts. A significant limitation of this study is the exclusion of non-English language papers.

## 6. Further Studies

As mentioned, due to a lack of reports on paraneoplastic syndromes, much of their pathophysiology remains unknown and is only speculated from empirical evidence or observations. The current existing evidence on the paraneoplastic symptoms of gallbladder cancer is primarily derived from case reports and should be further evaluated using large-scale cohort studies or even prospective studies. Further studies trying to establish the mechanism behind the manifestations of these syndromes, particularly the cross-reactivity with non-tumour cells, can aid in the development of effective targeted therapies. More research could also be conducted into identifying specific biomarkers that are associated with early paraneoplastic syndrome manifestations (i.e., autoantibodies in neurological paraneoplastic syndrome) allowing for quicker diagnosis and subsequent treatment.

## 7. Conclusions

This systematic review compiled PNSs associated with GBC, a relatively under-researched area. It contributes to the existing literature by synthesising and analysing different PNSs along with their management strategies and effectiveness. This study also investigates a broader population to include patients with gallbladder cancer in general instead of focusing on a type of cancer, which allowed for the identification of any patterns or unique findings in the studies. The risk of bias in each individual case report was also assessed to enhance data robustness compared to prior studies. As more cases connecting PNS to GBC are documented, future research could investigate the long-term effectiveness of treatments for both the cancer and the syndromes. Additionally, efforts could focus on developing methods to identify GBC earlier when PNSs appear, as well as gaining a deeper understanding of the underlying pathophysiological mechanisms linking these syndromes to GBC.

## Figures and Tables

**Table 3:** Studies reporting paraneoplastic Acanthosis nigricans and signs of Leser–Trélat associated with gallbladder cancer (GBC).

S/N	First Author, Year	Age/Sex	Country	Timing of PNS Diagnosis	Type of Cancer	Tumour Characteristic	Presentation	Investigations	Management	Survival Time After PNS Diagnosis	Study Bias
1	T. Ziadi, 2009 [65]	45/F	Morocco	BD	Adenocarcinoma	M	Mucocutaneous lesions on soles of feet, palms of hands, thighs, neck and armpits	Routine laboratory tests were normal	Gemcitabine Oxaliplatin	6 months	8
2	M. I. Jacobs, 1981 [69]	56/F	USA	BD ^a^	Adenocarcinoma	M ^c^	Abdominal pain Early satiety Nausea and frequent emesis Weight loss	High SGOT ^e^, SGPT ^f^, LDH ^g^, and CEA ^h^	5-fluorouracil Carmustine Doxorubicin	3 months	8
3	Ramirez-Amador V, 1999 [74]	55/F	Mexico	ATD ^b^	Adenocarcinoma	LA ^d^	Dysphagia Axillary, palpebra, and scalp hair loss Ulcers on palate Sweating and shivering Hyperpigmentation of forehead and neck Pruritus	Oral biopsy test weakly positive for HPV ^i^-35 in HPV PCR ^j^	5-fluorouracil Cisplatinum	Alive after 4 months	7

^a^ BD: before diagnosis of GBC, ^b^ ATD: at the time of diagnosis of GBC, ^c^ M: Metastatic, ^d^ LA: Locally advanced, ^e^ SGOT: serum glutamic-oxaloacetic transaminase, ^f^ SGPT: serum glutamate pyruvate transaminase, ^g^ LDH: lactate dehydrogenase, ^h^ CEA: carcinoembryonic antigen, ^i^ HPV: human papillomavirus, ^j^ PCR: polymerase chain reaction.

**Table 4:** Studies reporting paraneoplastic hypercalcaemia associated with gallbladder cancer (GBC).

S/N	First Author, Year	Age/Sex	Country	Timing of PNS Diagnosis	Type of Cancer	Tumour Characteristic	Presentation	Corrected Ca/mg/dL (Normal Range: 8.5–10.5 mg/dL)	Investigations	Management	Survival Time After PNS Diagnosis	Study Bias
1	Y Watanabe, 1989 [11]	54/F	Japan	ATD ^b^	Adenocarcinoma	M	Remittent fever Left hypochondric pain	17.6	Neutrophilia Normal PTH Normal cAMP ^g^ Low vitamin D High PTHrP	Normal saline, furosemide, indomethacin, prednisolone, and calcitonin mithramycin	47 days	8
2	M Takahashi, 1985 [34]	72/F	Japan	AD	Adenocarcinoma	LA ^d^	Right upper abdominal pain Nausea	15.4	Granulocytic hyperplasia Contrast-enhancing mass on CT Neutrophilia High PTHrp	Laparotomy with 5 mg of OK432 immunopotentiator was injected into the tumor	54 days	8
3	Masahiro Yanagi, 2023 [39]	71/M	Japan	ATD	Adenosquamous carcinoma	M	Right sided abdominal pain	14.9	High G-CSF ^h^, Neutrophilia High PTHrP	GEM ^i^ chemotherapy	27 days	8
4	Takahashi, K, 2024 [108]	43/F	Japan	ATD	Adenocarcinoma	M	Nausea Obstructive jaundice	15.5	High PTHrP Normal PTH	Saline infusion Bisphosphonate	6 weeks	7
5	M. Yogarajah, 2016 [110]	86/F	United States	AD ^a^	Adenocarcinoma	M ^c^	Weakness Loss of appetite, poor oral intake	15.5	PTH ^e^: 162 pg/mL (normal range 15–65) Normal PTHrP ^f^, 25-hydroxyvitamin D and 1,25-dihydroxyvitamin D levels Parathyroid scan: normal function, no lesion	IV hydration, calcitonin, IV pamidronate Cinnacalcet	Not stated (placed on palliative care)	7

^a^ AD: After diagnosis of GBC, ^b^ ATD: At the time of diagnosis of GBC, ^c^ M: Metastatic, ^d^ LA: Locally advanced, ^e^ PTH: Parathyroid hormone, ^f^ PTHrP: Parathyroid hormone-related protein, ^g^ cAMP: Cyclic adenosine monophosphate, ^h^ G-CSF: Granulocyte colony-stimulating factors, ^i^ GEM: Gemcitabine.

**Table 5:** Studies reporting paraneoplastic hyponatraemia associated with gallbladder cancer (GBC).

S/N	First Author, Year	Age/Sex	Country	Timing of PNS Diagnosis	Type of Cancer	Tumour Characteristic	Presentation	Serum Sodium (mmol/L)	Serum Osmolality (mmol/kg)	Urine Sodium (mmol/L)	Urine Osmolality (mmol/kg)	Investigations	Management	Survival Time After PNS Diagnosis	Study Bias
1	T. Tamura, 2013 [8]	47/F	Japan	BD	GBC	M	General fatigue	111	219	213	543	Serum ADH ^e^ 5.8 pg/mL (normal: 0.3–3.5 pg/mL) CT thorax: no pulmonary lesions Brain CT and MRI: no lesions Thyroid function: normal Adrenal function: normal	Sodium replacement, fluid restriction, radiotherapy Mozavaptan Resection of metastatic abdominal nodule	7 months	8
2	Esther S. Ng, 2010 [115]	35/F	Singapore	BD ^a^	Small-cell carcinoma	M ^c^	Epigastric pain radiating to the back Vomiting after meals Non-vertiginous giddiness	107	231	64	225	CT thorax: no pulmonary lesions Thyroid function: normal Short synacthen test: adequate rise in cortisol	NaCl tablets, fluid restriction Palliative chemotherapy (cisplatin, etoposide)	>275 days	7
3	Ayesha Saleem, 2016 [127]	72/F	Pakistan	ATD ^b^	Small-cell carcinoma	M	Jaundice Epigastric pain Lethargy and anorexia	128 (initial) 106 (after 4 months)	230 (after 4 months)	50 (after 4 months)	220 (after 4 months)	FBC: normal Serum K: 4.9 mmol/L Serum Ca: 10.2 mmol/L	Conservative management, fluid restriction first four months (NE) Hypertonic saline after 4 months	7 months	8
4	Gorrepati, V. S, 2022 [130]	54/F	USA	ATD	GBC	LA ^d^	Jaundice Nausea Fatigue	114	285	-	-	Hypokalemia of 2.9 mEq/L, and Hypochloremia of 79 mEq/L Serum glucose: 157 mg/dL Blood urea nitrogen: 44 mg/dL, Serum creatinine: 1.79 mg/dL	Cholestryamine and ursodiol	-	7

^a^ BD: Before diagnosis of GBC, ^b^ ATD: At the time of diagnosis of GBC, ^c^ M: Metastatic, ^d^ LA: Locally advanced, ^e^ ADH: Antidiuretic hormone. Serum Sodium normal range 135–150 mmol/L, Serum Osmolality normal range 285–295 mmol/kg, Urine Osmolality normal range 100 to 1200 mmol/kg, Urine Sodium normal range 40 to 220 mmol/L.

## Abbreviations

The following abbreviations are used in this manuscript:

GBC	Gallbladder cancer
PNS	Paraneoplastic syndromes
AN	Acanthosis nigricans
PTH	Parathyroid hormone
PTHrP	Parathyroid hormone-related peptide
G-CSF	Granulocyte colony stimulating factor
SIADH	Syndrome of Inappropriate Antidiuretic Hormone production
